# Teachers’ concerns about integrating information and communication technologies in the classrooms

**DOI:** 10.1371/journal.pone.0249703

**Published:** 2021-05-03

**Authors:** Opeyemi Dele-Ajayi, Oluwakemi Dunsin Fasae, Akachukwu Okoli

**Affiliations:** 1 Digital Learning Laboratory, Northumbria University, Ellison Place, Newcastle upon Tyne, United Kingdom; 2 Stemres Learning, Ikeja, Lagos, Nigeria; Universitá degli Studi di Bergamo, ITALY

## Abstract

Teachers in developing countries are facing increasing social and political pressure to use Information and Communication Technologies (ICT) to improve the access to and the quality of education available to young people. This is a core part of several government-led initiatives to attain the United Nations Sustainable Development Goal 4-quality education. While there is no shortage of ICT, the adoption for actual use in the classroom is often a hurdle for teachers, due to various concerns they harbour. This research study used the Concerns-Based Adoption Model (CBAM) to assess the stages of concern of 340 Nigerian teachers about adopting and integrating ICT in the classroom. The findings indicated that teachers’ concerns were most intense in the awareness, management and information stages respectively, and lowest at the collaborative and consequence levels. Further examination of the results also shows a significant relationship between the stages of concern and teachers’ personal attributes like teaching experience, age and the class level they teach. These findings provide practical insights into how to better create effective teacher professional development interventions, to assist teachers in adopting and integrating ICT, to enhance the learning experience of young people within the classroom.

## Introduction

A steady proliferation of ICT in everyday life has led to an increased interest in their application to education. As pandemics continue to cause disruptions to educational systems across the world, Educational Technology (EdTech) advocates and researchers predict that more learning and teaching will happen via ICTs now and in the coming years [1,2]. This study adopts the general definition of ICTs as “information and communication technologies that enable the production, storage and handling of information, and facilitate different forms of communication between human beings and electronic systems and among electronic systems in digital, binary computer language” [3]. Some of these ICTs include mobile and web applications, digital games, tablets, laptops, social media and mobile phones [4]. As young people increasingly get exposed to ICTs, the debate has moved from ‘should they be using them?’, to how educational benefits can be acquired from their use [5]. Numerous studies have established that ICTs are indeed useful for learning. Studies have shown that Web 2.0 technologies can also provide better learning engagement and support for students to learn actively and collaboratively [6]. Witt et al. [7] identified convenience (easy access), portability (anywhere access) and immediacy (anytime access) as benefits in their study of students’ use of tablets for learning. A handful of studies maintain that digital games have the potential to help learners inculcate 21st century skills like creativity, critical thinking, collaboration and communication [8,9]. In their study of the use of mobile digital games in the traditional classroom, Dele-Ajayi et. al. [5] found that young people who used mobile games as supplements in mathematics classrooms had a better learning experience and participated more than those who did not.

For many developing countries, the most important value of ICTs lies in its student-centric approach as it encourages the active participation of students in learning activities. In Nigeria for example, many classrooms are considered to be unengaging, boring and teacher-centred [10] with rote learning and memorisation of facts prevalent in classroom education. ICTs have the potential to improve the participation of young people by increasing their motivation and providing a more engaging classroom experience. Despite the advantages that ICTs offer within the traditional classroom; evidence suggests that it can cause disruption to the traditional classroom dynamics teachers are already used to [10]. This is a plausible explanation as to why classrooms across the world, especially those in developing countries are yet to fully experience the benefits that ICTs and EdTech offer [11]. Teaching professionals are the main drivers of change in the classroom and the adoption of ICTs is often heavily dependent on them. Likewise, the successful continued use is greatly determined by how well a teacher integrates ICTs for teaching and learning within the classroom [12]. While teachers are increasingly using ICTs in their daily lives, they have not adequately adopted these for use in the classroom [13]. Many studies have sought to understand the process of acceptance of ICTs by teachers using Technology Acceptance Models (TAMs) [14,15] and the Innovation Diffusion Theory [16].

In the specific context of developing countries, several studies have also explored the barriers to ICT adoption and integration. In Ghana, studies show that insufficient teaching technology expertise [17], lack of adequate training and access issues are barriers to teachers using ICTs in education [18]. Teachers in South Africa reported that unreliable electricity was a barrier to the use of ICTs for teaching in their classrooms [19]. Likewise, in Nigeria, teachers identified unstable educational curricula, poor orientation towards ICT and government policy as barriers to adoption in the classroom [13]. As majority of the schools in Nigeria are government owned, the practices in these schools are often influenced strongly by the policies of the government. According to a rapid scan of the EdTech space in Nigeria [20], the Nigerian government has an ICT in education policy [21] which was developed due to a need for coordination and standardization of the deployment of ICT in education. In addition to this policy, a National Implementation Guideline for ICTs in Education [22] was also developed to give clear instructions to education stakeholders across the country in the deployment of technology in education. The authors [20], based on their study, say that, very little progress has been made in putting into action these policies and guidelines as schools in Nigeria are still not equipped enough in the use of ICTs in the classroom. They also highlighted that data on state of ICT infrastructure in primary and secondary schools in Nigeria is not always readily available, majorly because the data were never collected, or it’s not made available to the public. They however, made their conclusions based on the statements from a representative of the ministry of education, who said, the technological situation in most primary and secondary schools is generally bad. Majority of the primary schools do not have access to electricity, internet access or computing devices for teaching or learning. The situation is fairly better in secondary schools where they are connected to electricity but with several weeks of blackout and when available, very low current. They often resort to the use of generators which are often restricted to specific places in the schools like the principals’ offices. The secondary schools, like the primary schools, do not have internet connectivity. Even though, there have been projects and initiatives carried out by the federal and state governments, private institutions and non-profit organizations, to improve access to ICTs in schools, like installation of solar panels to supply electricity to schools, donations of computers and laptops to schools, etc., there are still a lot of schools that do not have these infrastructures. For those who have, due to lack of maintenance and poor use of the infrastructure, they begin to fail. This situation in Nigeria, apart from the perception of teachers makes the adoption of technology in the classroom difficult.

Due to cultural, social and individual differences, teachers across the world perceive technology differently and their use of technology in education is strongly determined by how they think and feel about it [23]. Ertmer et. al. [24] broadly classified barriers to adopting and using technology under first and second order barriers. The first-order barriers are external: those related to resources e.g. access to technology, technical and administrative support and second-order barriers are internal: those barriers related to teachers and their attitudes e.g. beliefs about classroom practices and routines, unwillingness to embrace change and beliefs about teaching and learning. Ertmer et. al. [24] maintained that the second-order barriers often present a bigger challenge than the first-order barriers. Essentially, teachers are more likely to successfully navigate and tackle first-order barriers like lack of administrative and technical support if they have overcome second-order barriers like attitudes and belief about technology. In the process of integrating innovative ICT within classrooms, governments often use specialised professional development programmes as a means of addressing poor orientation and teachers’ skills gap in the use of technology [25]. It has been established that the willingness and ability of teachers to adopt and integrate ICTs in their classrooms for teaching is dependent on the quality of the professional development training they receive [23,26]. However, not all professional development programmes are effective as they often focus on skills acquisition rather than supporting the change process that teachers undergo in adopting and integrating innovation within the classroom [27]. In designing effective professional development programmes to support the change process, teachers’ concerns about making the change need to be accurately identified and addressed. A few studies have been conducted on teachers’ concerns and experiences about using ICTs and other innovations in the classroom in different parts of the world, mostly from western countries [28] where educational institutions are considered to be more matured and better positioned for technology adoption. To the best of the authors’ knowledge, this is the first study conducted into the concerns of schoolteachers about using ICT within the classroom in Nigeria. Nigeria has the largest population in Africa [29] and is currently dealing with a huge learning crises. A report by UNICEF [30] states that one in every five of the world’s out-of-school children is in Nigeria, with a total of 10.5 million children currently out of school [31]. There is a growing interest within governments and from funders towards the potential of ICT for addressing some of the learning crises [32,33]. Although research into the adoption and integration of technology is in its infancy [11] the authors hope that the results of this study would provide useful insights into how best teachers can be supported to use ICT in their classrooms.

## Theoretical framework

This study used the Concern-Based Adoption Model (CBAM), developed by [34,35] as a theoretical framework to understand the concerns of teachers about adopting ICT in their classrooms. The CBAM is based on an earlier work by Fuller [36] in which the concept of concerns was used to explore how training teachers responded to change at the start of their teaching careers. Teachers’ concerns about technology is a representation of their feelings, preoccupation, thoughts, consideration, and beliefs as regards the integration of technology in the classroom [36,37]. In introducing an innovation to teachers, their concerns could either facilitate or obstruct its implementation. The CBAM posits that change is not an event, rather developmental growth in feelings and skills, that occurs in stages. Teachers’ concerns changed over time from unrelated concerns (teachers are concerned about other things or are unconcerned about the innovation) to the focus on self (concern related to one’s self), to task (concern related to the implementation of the task) and finally to impact (concern related to the impact of the implementation on students’ learning in the classroom) [38]. These four categories are expanded into seven Stages of Concern (SoC):

### Unrelated concern

- Stage 0 (Awareness stage): In this stage, teachers have very little or no interest, awareness or involvement in the innovation.

### Self concern

- Stage 1 (Informational stage): In this stage, teachers have a general awareness and are seeking out more information about the innovation.
- Stage 2 (Personal stage): In this stage, teachers are concerned about the demands the innovation would place on their skills, their ability to meet the demands, and ultimately their role in the implementation of the innovation.

### Task concern

- Stage 3 (Management stage): In this stage, teachers’ concern and focus is on how to best implement the innovation and the management of the several operational aspects like resources, time and other duties.

### Impact concern

- Stage 4 (Consequence stage): In this stage, teachers focus on the impact of the innovation on their students’ learning.
- Stage 5 (Collaboration stage): In this stage, teachers focus on trying to understand how their colleagues are using the innovation and how they could coordinate, collaborate and cooperate with them.
- Stage 6 (Refocusing stage): In this stage, teachers focus is on exploring further ways to reap wider benefits from the innovation by modifying or innovating it.

Generally, with an innovation, a teacher often starts with higher levels of concern intensity on the self category (stages 1 and 2) stages. Through sustained use, they progress unto task (stage 3), and as the teacher becomes more experienced in the use of the innovation, they develop a higher intensity of concern over the impact categories (stages 4, 5 and 6). However, whilst these stages appear to evolve in this logical progression, they are not mutually exclusive as they often overlap, and a teacher could experience multiple stages of concern with different intensity levels at the different stages of concern. [39–41]. In order to measure the intensity of concern at the different stages, Hall et. al. [42] developed a 35-item diagnostic tool called the SoC questionnaire.

The SoC element of the CBAM has been widely used to explore the stages of concern of teachers in adopting an innovation across several countries. Most studies looked at the peak stage of concern, as well as the patterns of concerns exhibited by the entire population that was being studied. Hao et al. [40] studied 200 Taiwan teachers’ concern about integrating Web 2.0 technologies in education. They found that the teachers’ concerns were most intense at the informational stage, followed by the personal and management stages. Their findings suggest that most of the teachers in their sample study who had some level of awareness of Web 2.0 tools, were less worried about implementing the innovation but more interested in the general aspects of the technology in a less personal way. Liu et al. [43] examined the concerns of 86 in-service teachers in the United States about integrating technology. Their results also showed that teachers’ concerns were intense in the informational, personal and refocusing stages, suggesting that the teachers they studied had limited information related to technology integration even though they had access to internet in their classrooms. Similarly, in Ashrafzadeh et al. [39] study of 91 Iranian teachers, the peak of their concerns was shown to be at the information stage. Their study concluded that instructors need more information about how technology can be integrated into their teaching. Their study highlighted that anxiety with using technology in instruction is present even amongst experienced teachers who had previously received training on using ICT in the classroom. Jong [44] investigated the concerns of 118 teachers about using a Virtual Interactive Student-oriented Learning Environment (VISOLE) in school education. The teachers in their study had completed a VISOLE induction training before taking part in the SoC survey. Their results showed the teachers’ peak concern to be management, highlighting the support teachers would require to adopt and integrate VISOLE within their classrooms.

In addition to investigating the peak and pattern of concerns, these studies and some others have explored the relationship between stages of concern and teachers’ demographics or characteristics, with particular interest on gender, age and teaching experience. In their study of Taiwan teachers’ concern with integrating Web 2.0 tools for teaching, Chen et al. [41] found that across most of the stages, male and female teachers expressed similar intensity of concern. However, in the three internal stages (0, 1 and 2), females had significantly higher intensity than males. This finding led the authors to suggest that female teachers in their cohort were more anxious about using Web 2.0 applications than males or were less aware about technology integration for education. Their study further highlighted significant correlations between teachers’ stages of concern and some other characteristics like areas of discipline and self-reported levels of Web 2.0 use in teaching. However, Ashrafzadeh et al. [39] did not find any significant difference between teachers’ stages of concern and any of their demographic features–age, gender, field of study and teaching experience. In Al-rawajfih et al. [45] study of 350 Jordanian teachers’ concerns about integrating e- learning in schools, males and females did not show any significant difference in the intensity of concerns they expressed across the different stages. However, their study found a significant difference in teachers’ concern in relation to years of teaching, as teachers with less teaching experience expressed concerns about the collaborative stages of integrating e- learning while teachers with more years of experience had significantly higher personal concerns–on stages 0,1 and 2. The aforementioned studies indicate mixed findings from stages of concern studies. It is important to also note that majority of stages of concern studies are based in a specific and particular country or region as often times, the concerns of teachers are context specific. Although a general knowledge of teachers’ concerns across different contexts is useful, in order to effectively plan professional development programs to support teachers in a particular context, it is important to investigate their own concerns, taking into consideration the peculiarities and intricacies of their contexts. To date, few studies have empirically investigated instructors’ concern about integrating ICT in education in Nigeria, and none has looked specifically at primary and secondary school teachers. Only one study carried out by Igoche [46] explored the concerns of Nigerian instructors toward the implementation of ICT such as the internet and its tools for instructional purposes. This study carried out in 2010 investigated university level instructors that held the ranks of lecturers, senior lecturers, associate professors and professors. As calls increase for primary and secondary school teachers to adopt ICTs for teaching, it is important to understand what their concerns are and create effective programmes to support their professional development. Therefore, our study investigated teachers’ concerns shown in Table 1 about using ICTs in the classroom and the relationship between this and teachers’ characteristics. The following research questions were explored during the study:

1. What are teachers’ concerns about the use of ICT in the classroom?
2. What are the relationships between teachers’ stages of concern and teachers’ characteristics?

**Table 1:** Typical expressions of concern about an innovation.

Categories	Stages	Expression of Concern
Unconcerned	0	I am not concerned about it.
Self	1	I would like to know more about it
	2	How will using it affect me?
Task	3	I seem to be spending all my time getting materials ready
Impact	4	How is my use affecting my students?
	5	I would like to coordinate my effort with others, to maximize the innovation’s effect.
	6	I have some ideas about something that would work even better.

## Materials and methods

This research received ethical clearance from Northumbria University and Ekiti State Ministry of Education, Science and Technology. Participants read information sheets and signed appropriate consent forms before the research started. Participation was voluntary. The ethics submission reference number is 17412.

### Participants

The participants were primary and secondary school teachers from several schools in Ekiti, a state in southwest Nigeria. Nigeria’s education system has 3 sectors: Basic education (9 years: 6 years of primary education usually for children of ages 6–11 and 3 years of junior secondary education for children of ages 12–14), Post basic/senior secondary education (3 years for children between ages 15–17) and Tertiary education (4–6 years). Ekiti has 16 local governments. Data was gathered from a convenience sample of 500 teachers that attended a 3-day workshop on “EdTech in Schools” and sponsored by the World Bank State Education Programme Investment Project (SEPIP) in Ekiti State. The aim of the workshop was to expose teachers to the value of EdTech and the roles ICT can play in education. The teachers to attend the workshop were randomly selected by their schools across all classes and across all subjects. A breakdown of the sample statistics of teachers in this study is shown in Table 2. The teachers filled the SoC questionnaire (S1 File) on the first day of the workshop, before any form of training or seminar was delivered by the facilitators. Amongst the 500 participants, 340 completed the survey. Data of 160 teachers was not included in the analysis because of incomplete or empty responses.

**Table 2:** Teacher sample statistics.

		Freq.	Perc (%)
**Gender**	Male	135	39.7
	Female	205	60.3
**Age**	<20 years	5	1.5
	20–30 years	213	62.6
	31–40 years	78	22.9
	41–50 years	34	10.0
	Over 50 years	10	2.9
**Digital competence level**	Poor	0	0
	Moderate	199	58.5
	Good	116	34.1
	Very good	24	7.1
**Years of teaching**	< 1 year	38	11.2
	1–5 years	120	35.3
	6–10 years	115	33.8
	11–20 years	42	12.4
	Over 20	25	7.4
**Level of class taught**	Primary school	181	53.2
	Secondary school	159	46.8

### Data collection

In order to measure Nigerian teachers’ stages of concern, we adopted the original SoC questionnaire [47]. The term ‘innovation’ used in the original questionnaire was changed to ‘ICT’ in the revised questionnaire that was used with teachers. The SoC questionnaire [47] has been widely adopted and used for teachers in several studies [48–50] to identify the intensity of the seven stages of concern associated with the introduction of an innovation—usually technology and computing. Each of the seven stages contains five items, making a total of 35 items using an 8-point Likert scale from 0 - “not true of me now” to 7- “very true of me now”. As a widely used tool, several studies have confirmed the validity and reliability of the instrument using different strategies like focus groups and interviews [40] However, the researchers carried out further reliability analysis on the revised questionnaire as seen in Table 3 which shows the stages of concern with the corresponding reliability and sample items in each stage. In addition to this, two EdTech researchers and two teachers in Nigeria checked the revised questionnaire for construct validity and appropriateness of language.

**Table 3:** Stages of concern reliability scores.

Stage	Cronbach’s alpha	Sample items
**0(Awareness)**	0.88	I spend little time thinking about the use of Information and Communication Technologies in the classroom
**1(Information)**	0.84	I would like to know how the use of ICT is better than what we have now.
**2(Personal)**	0.81	I would like to know how my teaching or administration is supposed to change.
**3(Management)**	0.86	I am concerned about my inability to manage all that ICT require.
**4(Consequence)**	0.82	I am concerned about how ICT affects students
**5(Collaboration)**	0.83	I would like to help other teachers in their use of ICT.
**6(Refocusing)**	0.83	I would like to determine how to supplement, enhance, or replace ICT
**SoC Total items**	**0.83**	

### Data analyses

**Fig 1:**
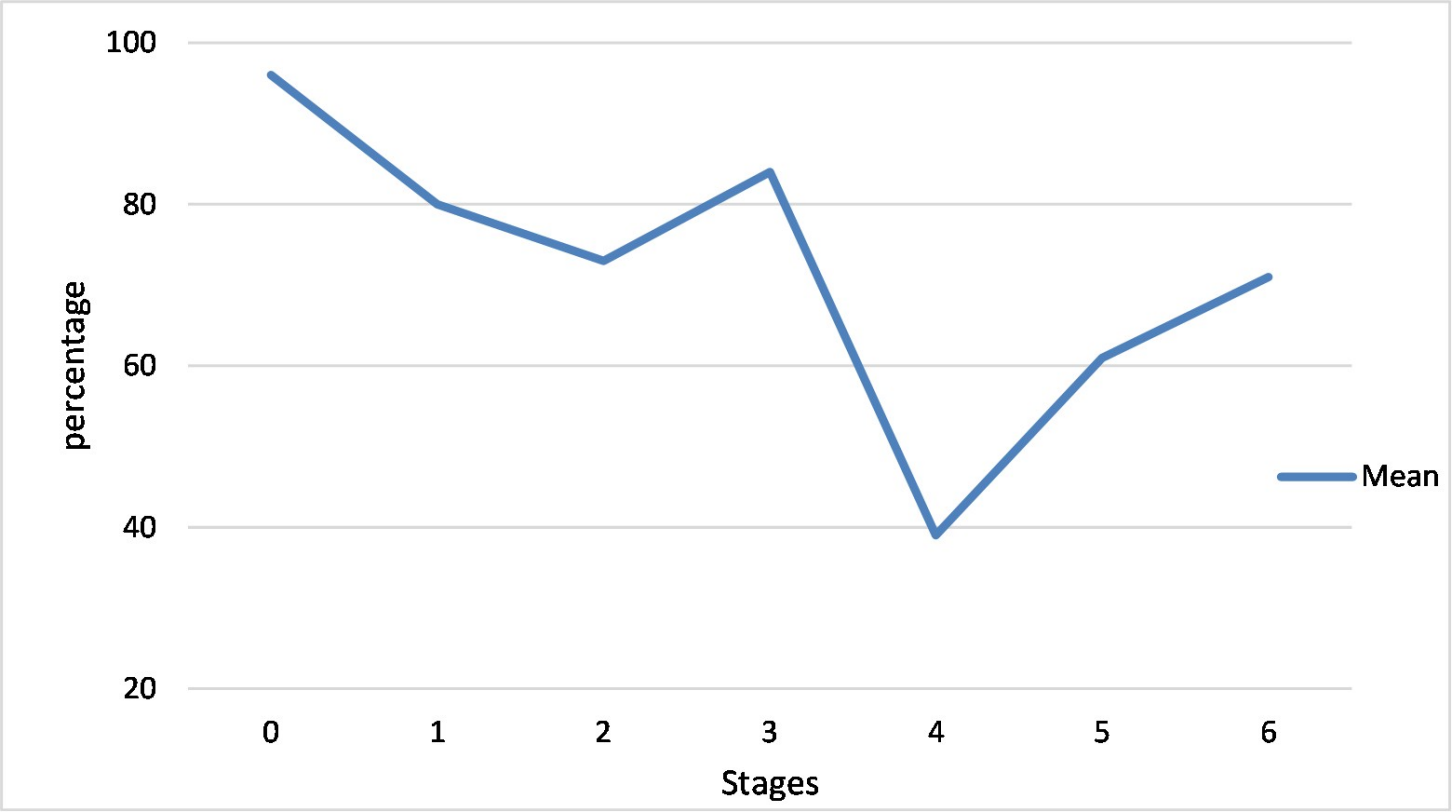
SoC percentile scores for total group.

The SPSS version 19 software was used in the analysis of the data collected. Following guidance from the implementation guidelines by Hall et al. [42], the descriptive raw scores for each subscale in the SoC results were summed and converted to percentile scores using the percentile conversion chart for the SoC questionnaire. Following Yean et al. [51] results from examining trends and patterns from the SoC are more valid than results from analysing statistical significance amongst the stages. Therefore, (Fig 1) shows a graphic representation of the pattern for SoC percentile scores for the total group. The graph answers the first research question–what Nigerian teachers’ concerns are about using ICT in the classroom.

To answer question 2 –“can personal characteristics predict teachers’ stages of concern about adopting ICT in the classroom?”, the researchers tested the group difference, and also performed t-tests and one-way ANOVA. Teacher attributes like gender, age, years of teaching, experience with the use of ICT, subject taught and the level of class taught were analysed.

## Results and findings

Table 4 presents the descriptive statistics for SoC subscales for Nigerian teachers (n = 340).

**Table 4:** Stages of concern statistics.

Stage	Mean	Std. Dev.	Minimum	Maximum
0(Awareness)	95.81	5.37	61.00	99.00
1(Information)	79.59	13.97	43.00	99.00
2(Personal)	72.99	17.54	41.00	96.00
3(Management)	83.75	9.80	30.00	99.00
4(Consequence)	38.68	22.07	5.00	90.00
5(Collaboration)	61.37	23.81	14.00	95.00
6(Refocusing)	71.09	16.69	30.00	99.00

The following subsections present the findings from the research questions.

**Fig 2:**
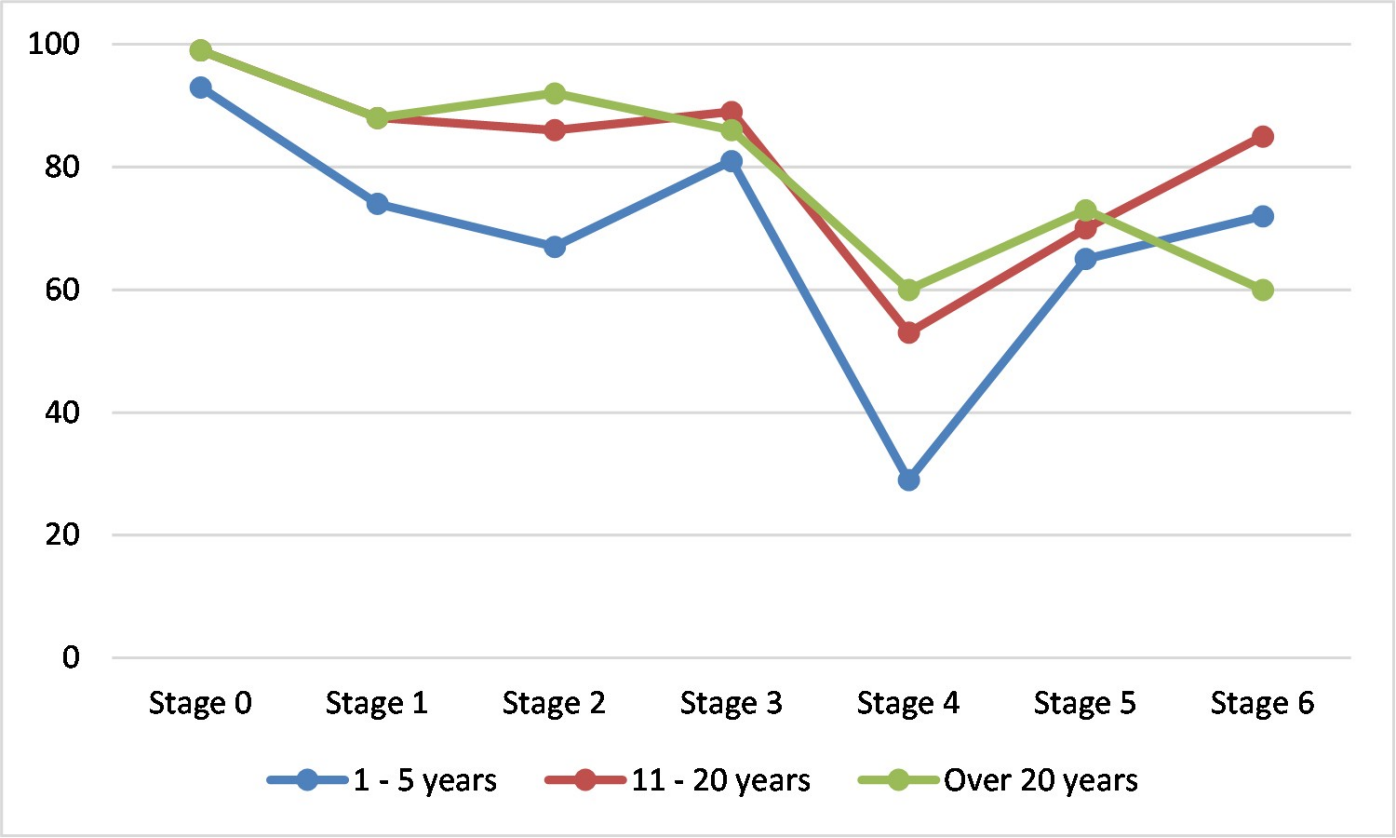
SoC intensity according to years of teaching experience.

- ***What are teachers’ concerns about the use of ICT in the classroom***?
  The teachers had the highest concern mean percentile of 95.81% in Stage 0 (awareness) and the least concern of 38.68% in Stage 4 (consequence). (Fig 1) shows that the teachers focused their concern in stage 0 followed by stage 3 (management) and then stage 1 (information). Teachers had similar concern levels in stage 2 (personal) and stage 6 (collaboration) and then lower concerns in stage 5 after which there was a significant drop to stage 4 (consequence).
- ***Teachers’ characteristics and its relationship with their stages of concern***.
  While gender made no statistically significant difference, other teacher characteristics like teaching experience, age, experience with the use of ICT, and the level of class taught made significant differences in the stages of concern. The numbers of years spent in teaching made a statistically significant difference on the level of concern the teachers expressed across all the stages of concern as determined by one-way ANOVA at a 95% confidence level. The mean score intensity for each group is plotted across all the stages to show the pattern of concern expressed by the teachers based on the years of teaching experience they have as shown in (Fig 2).

A Tukey post hoc test, the results shown in Table 5, revealed that: the mean concern intensity of teachers with 1–5 years of teaching experience (M = 92.59, SD = 5.53) at stage 0 (awareness) was statistically significantly lower at a significant level of 0.05than the mean concern intensity of teachers who had 6-10years of experience (M = 96.80, SD = 5.59). Also, at this stage, there was no statistically significant difference between the concern levels of teachers with other years of experience.

**Table 5:** Results of One-way ANOVA for the effect of years of teaching on the concern intensity of teachers across stages.

Stage	Groups	Mean	SD	ANOVA result (At a significant level of 0.05)
**0 (Awareness)**	1–5 years	92.59	5.53	F (4,335) = 22.76, p = 0.000
	6-10years	96.80	5.59	
**1 (Information)**	1-5years	74.36	16.06	F (4,335) = 11.31, p = 0.000
	Over 20years	88.16	12.67	
**2 (Personal)**	6–10 years	72.11	16.49	F (4,335) = 21.20, p = 0.000
	11–20 years	85.79	15.12	
**3 (Management)**	1–5 years	81.14	10.78	F (4,335) = 5.45, p = 0.000
	11–20 years	88.76	7.71	
**4 (Consequence)**	6–10 years	38.23	17.91	F (4,335) = 19.37, p = 0.000
	11–20 years	53.40	17.19	
**5 (Collaboration)**	<1year	42.39	28.52	F (4,335) = 11.15, p = 0.000
	Over 20 years	72.92	13.97	
**6 (Refocusing)**	Over 20 years	59.92	16.08	F (4,335) = 13.00, p = 0.000
	11–20 years	84.64	13.51	

Teachers with 1–5 years of experience had statistically significant lower concerns at a level of 0.05 in the information stage (stage 1) (M = 74.36, SD = 16.06) compared with teachers with over 20 years of experience (M = 88.16, SD = 12.67). There was no statistically significant difference between other groups at stage 1.

At stage 2 (personal) and stage 4 (consequence), teachers with 11–20 years of experience had significantly higher mean score concerns (M = 85.79, SD = 15.12) at a level of 0.05 than teachers with 6–10 years of experience (M = 72.11, SD = 16.49). There was no statistically significant difference in the concern levels of teachers in the <1, 1–5 and over 20 groups at these stages. In stage 3 (management), teachers with 1–5 years of teaching experience had significantly lower concerns (M = 81.14, SD = 10.78) than teachers with 11–20 years (M = 88.76, SD = 7.71). There was no significant difference in the concern intensity of teachers in the other age groups.

In stage 5 (collaboration), teachers with over 20 years of teaching experience had significantly higher concern intensity (M = 59.88.14, SD = 10.79) than teachers with less than 1-year teaching experience. There was no significant difference in the concern intensity of other age groups in stage 5 (collaboration).

Teachers with 11–20 years of teaching experience had significantly higher concern intensity in stage 6 (refocusing) (M = 84.64, SD = 13.52) than teachers with over 20 years’ teaching experience (M = 59.92, SD = 16.08) at a confidence level of 0.05.

There was no significant difference between the concern intensity of other groups of teaching experience.

Gender had no significant difference in the intensity of the concern in any of the stages. Age of teachers on the other hand had significant impact on the levels of concern they expressed at each stage. Repeated measures ANOVA tests were performed on each of the seven stages of concern at 95% confidence level. and the ANOVA results show that the age of a teacher had an effect on the intensity of concern across all the seven stages.

**Fig 3:**
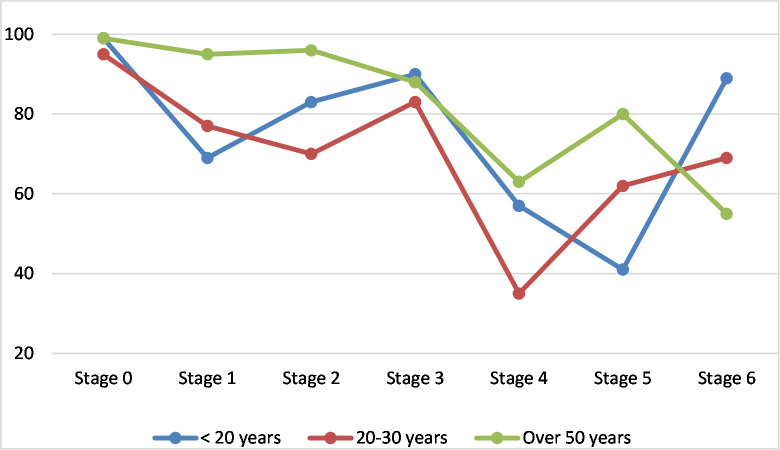
SoC intensity according to age.

To identify the age groups that made significant differences across the stages, a pairwise Tukey post hoc comparison was conducted and the results shown in Table 6 revealed that: age didn’t have any effect on the level of concerns of teachers at stage 0 and stage 3; teachers of more than 50 years had significantly higher concerns (M = 95.00, SD = 0.00), (M = 80.00, SD = 0.00) than those who were less than 20 years (M = 68.80, SD = 6.26), (M = 40.60, SD = 7.47) in stages 1 (information) and 5 (collaboration) respectively. Teachers in mid age groups had similar intensity levels with no significant difference. Also, in stage 2 (personal) and stage 4 (consequence), teachers within age group 20–30 had the lowest mean concern intensity (M = 69.92, SD = 16.81), (M = 35.07, SD = 22.43) while teachers of more than 50 years of age had highest concern intensity (M = 96.00, SD = 0.00), (M = 63.00, SD = 0.00) respectively. In stage 6 (refocusing), there was a different pattern as teachers of less than 20 years of age had the highest mean concern intensity (M = 88.60, SD = 15.32) and teachers with over 50 years of age had the lowest concerns (M = 54.50, SD = 2.63). The pattern of teachers’ concerns influenced by age across all the stages is shown in (Fig 3).

**Table 6:** Results of One-way ANOVA for the effect of age on the intensity of teacher’s concern across all stages.

Stage	Groups	Mean	SD	ANOVA result (At a significant level of 0.05)
**0 (Awareness)**	No significant difference			F (4,334) = 6.59, p = 0.050
**1 (Information)**	< 20years	68.80	6.26	F (4,334) = 10.47, p = 0.000
	Over 50 years	95.00	0.00	
**2 (Personal)**	20–30 years	69.92	16.80	F (4,334) = 9.97, p = 0.000
	Over 50 years	96.00	0.00	
**3 (Management)**	No significant difference			F (4,334) = 3.54, p = 0.08
**4 (Consequence)**	20–30 years	35.07	22.43	F (4,334) = 10.23, p = 0.000
	Over 50 years	63.00	0.00	
**5 (Collaboration)**	< 20 years	40.60	7.47	F (4,334) = 4.11, p = 0.003
	Over 50 years	80.00	0.00	
**6 (Refocusing)**	Over 50 years	54.50	2.635	F (4,334) = 9.238, p = 0.000
	< 20 years	88.60	15.32	

In our study, the teachers were asked to rate themselves based on how often they used digital devices personally and how convenient and confident they were in handling the devices. They were asked to choose from the options: poor, moderate, good and very good. From their response, none of the teachers felt poor about handling or using digital devices personally. Their responses were between moderate, good and very good, which is shown in the Table 2. The results determined with a One-way ANOVA at a confidence level of 0.05 showed that the level of experience and confidence of a teacher in using digital devices personally, had a statistically significant effect on the intensity of concern expressed at stage 0 (awareness, F (2,336) = 5.096, p ≤ 0.05), stage 1 (information, F (2,336) = 3.639, p ≤ 0.05), stage 2 (personal, F (2, 336) = 6.006, p ≤ 0.05), stage 5 (collaboration, F (2,336) = 13.072, p ≤ 0.05) and stage 6 (refocusing, F (2,336) = 17.563, p ≤ 0.05).

A Tukey post hoc analysis revealed significant differences in stage 0 (awareness) and stage 1 (information) where teachers who had moderate experience with the use of digital devices had significantly higher concern (M = 96.55, SD = 3.841), (M = 80.92, SD = 11.36) levels than those who had very good experience (M = 93.63, SD = 7.94), (M = 73.25, SD = 15.21) respectively. In stage 5 (collaboration), teachers who had good knowledge of the use of digital devices showed higher concerns (M = 69.89, SD = 20.16) than those with moderate knowledge (M = 56.17, SD = 24.75) and in stage 6 (refocusing), teachers with very good experience with use of digital devices had highest concerns (M = 83.08, SD = 15.89) than those with moderate level of use (M = 67.03, SD = 15.09). There was no significant difference within the groups in stages 2, 3 and 4.

**Fig 4:**
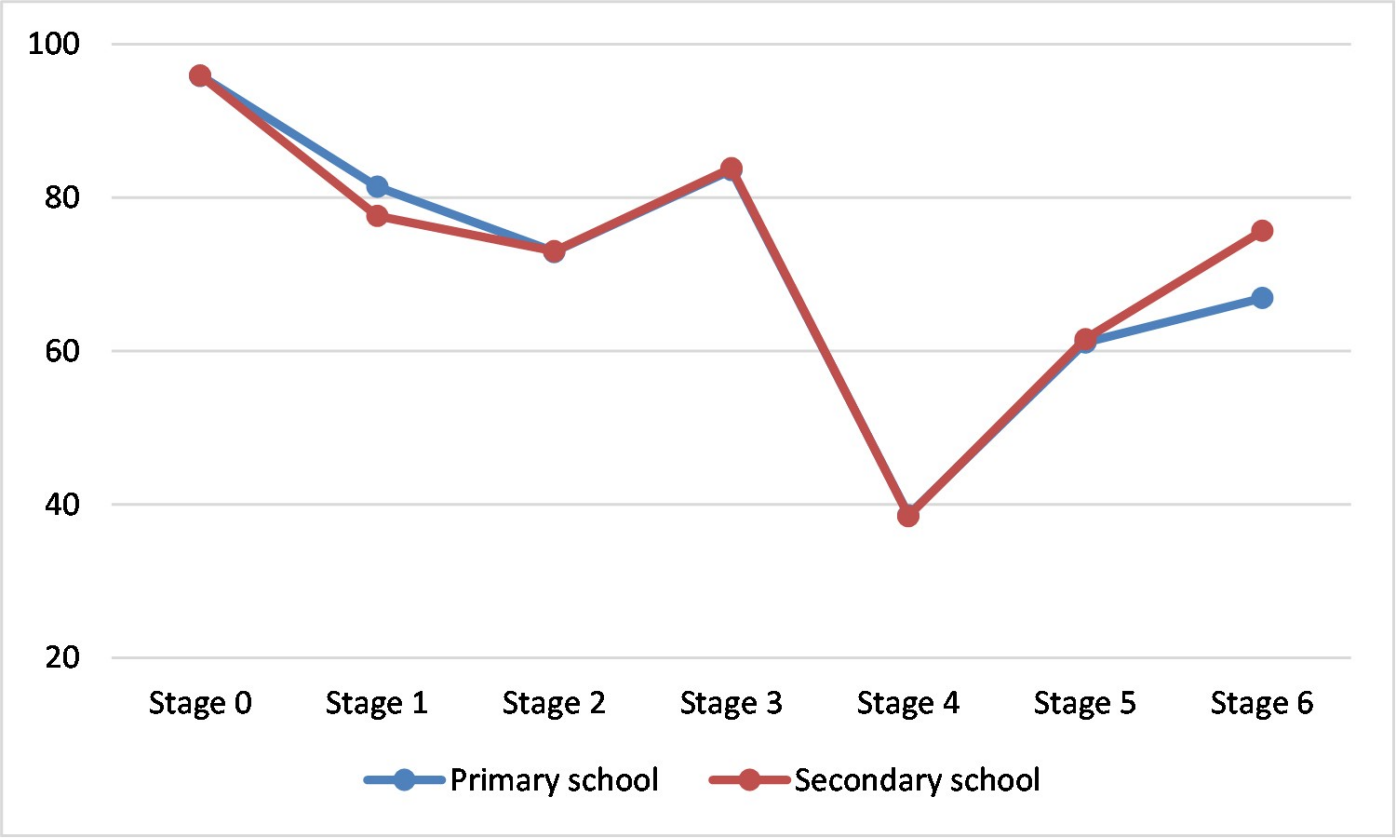
SoC intensity according to level of class taught.

In terms of the level of class taught, divided into primary and secondary classes, an independent samples t-test conducted revealed that the level of class the teacher taught had an effect on the level of concern observed in stage 1 (informational, t (307.31) = 2.49, p ≤ 0.05) and stage 6 (refocusing, t (337) = -4.99, p ≤ 0.05). Teachers who taught primary school classes had a higher intensity of concern (M = 81.43, SD = 12.64) in stage 1 (informational) than teachers who taught secondary classes (M = 77.62, SD = 15.11) while in stage 6 (refocusing), teachers who taught secondary classes had higher concern levels (M = 75.71, SD = 15.83) than those who taught primary classes (M = 66.94, SD = 16.36). This is depicted in (Fig 4).

## Discussion

According to the results and findings of the patterns of concerns of the teachers in this study, stage 0 (awareness) is the peak concern. This is an extremely rare finding as most of the previous studies do not show peak concerns in stage 0. This finding suggests that teachers in this study are either unconcerned or unaware about the use of ICT in education. Additionally, the high intensity in stage 1 (informational), stage 2 (personal) and stage 3 (management) is a typical pattern of self-focused concerns [52]. Based on the guidelines contained in Measuring Stages of Concern about the Innovation [47], the overall profile indicates that teachers in this study are nonusers of the innovation, which means they don’t use ICT in the classroom (Note that this is distinct from the general use of ICT in daily activities). Concerns of nonusers are normally highest on stages 0 (awareness), 1 (informational), and 2 (personal) and lowest on stages 4 (consequence), 5 (collaboration), and 6 (refocusing). The high score in stage 0 (awareness) demonstrates teachers’ degree of interest in and engagement with the use of ICT in education in comparison to other tasks, activities and efforts. It suggests that the use of ICTs in the classroom is not central to the thinking and practice of the teachers studied at the moment. This indicates that the teachers prioritize other tasks, initiatives and activities over adopting ICTs in the classroom. Stage 1 (informational) concern is relatively high and also suggests that the teachers are more likely to be unaware of the role of ICTs in the classrooms than unconcerned. This is also due in part to the patterns of concern not being consistent with the typical unconcerned innovation user in the measuring guidelines for the SoC [47]. The high scores in stages 0 and 1 is consistent with other studies that have shown that during the initial phases of the introduction of an innovation, teachers often demonstrate more intense concerns in the early stages [39–41]. Although the high scores in stage 1 (informational) do not confirm the current level knowledge of the teachers, it suggests they are interested in knowing more about the use of ICTs in their classrooms [39]. An interesting finding in this study is the relatively high stage 3 concern of the teachers, even though majority of the teachers in this study are nonusers. This is consistent with Hall [53] who suggests that high intensity management concerns are often observed amongst inexperienced users of an innovation. The relatively intense stage 3 (management) scores of the teachers in this study thus illustrates their concerns about time, logistics, and other management aspects teachers envisage will be a challenge to adopting ICTs within the classroom.

This study shows some significant correlations between teachers’ stages of concern and their demographics. For example, teaching experience made significant differences in all the stages which is consistent with [45]. In stage 0 (awareness), teachers with 1–5 years of teaching experience had lower concern intensity than teachers with 6 and more years of teaching experience. This suggests that experienced teachers were more likely to show little concern about or involvement with the use of ICT in the classroom. The higher concern intensity of more experienced teachers in stage 2 (personal) and stage 4 (consequence) indicates that teachers in this group are uncertain about the effects the use of ICT in the classroom might have on them, they are worried about being capable enough to handle the resultant changes and if they would be able to meet the demands due to ICT usage. They are also concerned about the impact this would have on students in the classroom. They worry about the relevance of ICT and if it will have the desired effects on the students’ learning outcomes, performances and competencies. They are also worried about the changes they might have to make, due to the adoption of ICT within the classroom in order to improve the students’ performance. Teachers with 11–20 years of teaching experience also had highest concerns in stage 3 (management) which indicates that the teachers were also focused on the processes and tasks involved in the use of ICT in the classroom and worry about being able to be organised and efficient in their duties while making use of ICT. They are also concerned about being able to manage their time and still achieve their set teaching goal. Teachers with 1–5 years of teaching experience were however, the least concerned about management. These findings contradict other studies [25,40] that showed no significant different between the years of experience and the stages of concern of teachers.

At stage 5, teachers with over 20 years of teaching experience had the highest concern intensity while teachers with less than 1-year teaching experience had the lowest concern intensity. This implies that teachers who have spent the highest number of years in teaching might be more interested in coordinating and cooperating with others in the use of ICT in the classroom while teachers with less than 1-year teaching experience are the least concerned about this. While teachers with over 20 years of teaching experience are interested in cooperating with others in the use of ICT in the classroom, they are however less concerned about exploring ways to make major changes to it or replacing it with a more powerful alternatives compared with teachers with less experience.

Age also made significant differences contradicting previous similar studies [39,40]. Significant differences were evident between age groups in only stage 1 (informational), stage 2 (personal), stage 4 (consequence) and stage 6 (refocusing). The high concern intensity of teachers of more than 50 years of age in stage 1 (information) and stage 5 (collaboration) indicates that the oldest group of teachers are interested in finding more information about the use of ICT in the classroom, they are also interested in coordinating and cooperating with others in the use of ICT in the classroom indicating an interest to leverage on their colleagues’ shared knowledge and expertise. This finding is consistent with the level of intensity teachers with over 20 years of teaching experience showed in stage 1 and stage 5, as older teachers would have more teaching years of experience. Teachers aged over 50 years also had high concern intensities in stage 2 and stage 4 which indicates that they are also worried about the effects of using ICT in the classroom on themselves and on the students.

The experience of the teachers in the use of digital devices showed significant differences in stage 0 (awareness), stage1 (information), stage 5 (collaboration) and stage 6 (refocusing). Teachers who had moderate experience with the use of digital devices had higher concern levels than those who had very good experience in stage 0 (awareness) and stage 1 (information) which indicates that the teachers who do not make use of digital devices often, are not less concerned about the use of ICT in the classroom but are interested in knowing more generally about the ICT that can be used in the classroom and how to use them. Teachers who use digital devices fairly regularly, showed higher concerns in stage 5 and stage 6 than those who do not use digital devices often. This indicates people with more experience in the use of digital devices, are willing to coordinate and cooperate with others in relation to the use of ICT in the classroom and are also interested in exploring ways to reap more universal benefits in the use of ICT in the classroom and are also willing to make suggestions to do things differently. This is consistent with the findings of [40] that suggests that concerns of teachers who are more skilled in technology were usually less ego-centric.

The relationship between the class level taught and their concern levels had not been studied before in literature. The teachers in this study teach in both primary and secondary schools. The results indicate that teachers at both levels had similar intensity of concerns in all stages apart from stage 1 (informational) and stage 6 (refocusing). In stage 1, primary school teachers had significantly higher informational concerns than secondary school teachers. This indicates that primary school teachers are less aware of the use of ICT in the classroom compared to secondary school teachers and are more concerned about learning about ICT. In stage 6, secondary school teachers had significantly higher levels of concern than primary school teachers. This higher stage 6 concerns indicate a stronger level of opinion on ICT. While these opinions are not necessarily positive or negative, the significantly higher intensity they demonstrate indicates a stronger exploration of ICTs to obtain more benefits, modifying the current approach to the use of ICTs or a complete replacement with alternative innovations. The results of this study indicate that males and females exhibited similar intensity of concerns across all the stages with no significant differences. Although this is consistent with some previous studies [39,45], others like [40] found differences in male and female stages of concern.

The overall SoC results show that despite the different effect teachers’ characteristics have on their intensity of concerns, majority of the teachers are currently unaware/unconcerned about the use of ICT in education. The overall findings confirm that despite its increasing penetration and adoption, the use of ICT in Nigerian education is still in its infancy and the support offered to teachers should reflect this reality. These findings present a number of implications for the design of teachers’ professional development programmes.

## Recommendations and limitations

As mentioned earlier on, in order to design effective teacher professional development programmes that empower teachers to effectively use ICT in the classroom the concerns of teachers need to be accurately identified and addressed. This study highlights the fact that Nigerian teachers have little or no awareness about the use of ICT in education. The results also suggest that teachers are currently more concerned about other activities and tasks. As highlighted by teachers in Nigeria in [13], this may be due to the evident barriers to the adoption of digital technologies in the classroom such as instability of the government, government policies, poor infrastructure and conducive learning environment due to underfunding of the education sector, poor electricity supply and internet connectivity. It also suggests that teachers’ poor salary, could be another factor which affects their perception to the adoption of technology, as they are more concerned with being paid better salaries than teaching with technology. Incentives and better salaries could motivate them to develop an interest in using the innovation. It therefore appears that professional development efforts that focus on particular digital skills, tools, and technologies are unlikely to be effective at the start. Consequently, the main goal would be to address teachers’ concerns at stage 0 (awareness), starting them on the right path to transitioning unto other stages. Following [53], one intervention to address stage 0 concerns is to share some information about the proposed ICT with a view to raising the interest and awareness of teachers. Professional development programs may develop and share resources and information to sensitize teachers about current ICT and the potential roles this plays in the traditional classrooms. If specific information is available about other issues teachers are concerned about, designers of teacher professional development programs may be able to relate these to the use of ICT and how it could be of benefit in this regard. For example, teachers that are concerned about improving the engagement of their students in the classroom may show more interest and willingness to use digital educational games if they are aware of its potential benefits in addressing their concerns. The strategies for developing effective professional development programs that address high intensity stage 0 concerns are also suitable for addressing stage 1 concerns. Hall [53] further suggests interventions that provide information contrasting what teachers are doing with what they would be doing when they adopt the innovation. In addition to this, using various ICT in existing professional development programmes will assist teachers in addressing the self-level concerns and gain useful information on how to successfully achieve integration [24,40].

Hall [53] provided guidance on addressing high intensity in stage 3 (management) concerns. Given that this identifies inexperienced users, professional development efforts should include adequate and comprehensive ‘how-to’ guidance for the use of ICT within classrooms. It is also important to demonstrate the importance of the use of ICT within current educational activities, and to highlight that it is not just an ‘add-on’. Creating a community of practice where teachers ‘buddy-up’ or share both successful and unsuccessful experiences of using ICT could also prove beneficial. Finally, asking expert teachers who have experience with using ICT to teach or train upcoming and inexperienced teachers could also be advantageous. This is in contrast to inviting digital technology experts who have no experience of teaching in the classroom to handle professional development programmes for teachers.

Although the findings generated from this study are insightful and provide useful implications for supporting teachers’ professional development, they are not without limitations. First, the findings discussed are from a relatively small sample of teachers from a state in a developing country, and also due to teachers’ response rate of 68%, the results should not be generalised to other contexts, especially in developed countries, where teachers may likely be more used to using ICT in the classroom. A further study is encouraged to specifically compare results from developed countries with those in developing countries to see if these are different and what knowledge can be acquired from the differences. Secondly, this study grouped all educational innovations into a single category—ICT. It is possible that different technologies like mobile phones, apps, laptops and websites may create different levels and intensity of concern to teachers. Future studies could investigate different ICT and examine if teachers express different levels of concern with these. Whilst the sample collected represents data from a state in Nigeria, based on the uniformity of the teaching curriculum and teacher training programmes used across the country, it is assumed that the teachers in the study are a fair representation of teachers in Nigeria, and adequately reflects the challenges and concerns expressed by teachers across the country in relation to the adoption of technology within the classroom. Further studies could investigate from a wider range of teachers across the country to compare the changes in the results if there exists. Finally, teachers’ concern in this study was before any introduction to the use of digital tools in the classrooms. The level and intensity of their concerns are expected to change as they become more aware and use ICT more in their classrooms. It will be important to study their concerns when this happens so as to adjust the professional development programmes to reflect the transitions and better support them to maximise the use of the ICT in their classrooms.

## Conclusion

Evidence from literature suggests that despite enormous investment in digital innovations for education globally, most of the technologies do not get adopted in the classroom [54]. One of the reasons why this is the case is that teachers’ concerns about adopting and integrating the technologies are often not identified and addressed [52]. Using the SoC element of the CBAM, this study investigated the concerns of 340 teachers with using ICT in their classrooms. It found that most of the teachers had the highest level of concern in Stage 0. Concerns in stage 0 relates to awareness of and little or no involvement with the use of ICT in the classroom. In general, teachers appear not to currently know the role ICT play in education. Furthermore, teachers’ characteristics like class level taught, experience with digital devices, age and experience of teaching had significant effects on the intensity and stages of concern. The findings from this study provide useful insights for policy makers and designers of teachers’ professional development programmes. The understanding of what the concerns of teachers are with adopting ICT for teaching in their classrooms can significantly increases the chances of successfully creating customised and delivering tailored support to teachers. It is hoped that addressing these concerns will facilitate better adoption and integration of ICT in the classroom, thus helping teachers and students benefit from the various advantages of technology- enabled learning.

## Supporting information

S1 File Stages of concern questionnaire. (PDF)

S2 File Stages of concern by teachers dataset. (XLSX)
