# Supplementary material for: Teachers’ concerns about integrating information and communication technologies in the classrooms
Source: PLoS One. 2021 May 3;16(5):e0249703. doi: 10.1371/journal.pone.0249703 (PMC8092759; doi:10.1371/journal.pone.0249703)
Supplement: S1 File — (PDF) [file pone.0249703.s001.pdf]

Stages of Concern Questionnaire

The purpose of this questionnaire is to determine what you are concerned with respect to the use of digital tools in your classroom.

The items (35) were developed from responses of school and college teachers who ranged from no knowledge at all about various innovations to many years' experience using them. Therefore, some of the items on this questionnaire may appear to be of little relevance or irrelevant to you at this time.

For the completely irrelevant items, please circle '0' on the scale. Other items will represent those concerns you do have, in varying degrees of intensity, and should be marked higher on the scale.

For example:

This statement is very true of me at this time.	6 7
This statement is somewhat true of me now	3 4 5
This statement is not true of me at this time.	1 2
This statement seems irrelevant to me now	0

Please respond to the items in terms of your present concerns, or how you feel about your involvement with digital tools in the classroom.

Thank you for taking time to complete this questionnaire.

0	1 2	3 4 5	6 7
**Irrelevant**	**Not true of me now**	**Somewhat true of me now**	**Very true of me now**

Circle One Number for Each Item

I am concerned about students' attitudes toward digital tools in the classroom.	0	1	2	3	4	5	6	7
I now know of some other approaches to teaching that might work better than using digital tools.	0	1	2	3	4	5	6	7
I am more concerned about another approach to teaching.	0	1	2	3	4	5	6	7
I am concerned about not having enough time to organize myself each day.	0	1	2	3	4	5	6	7
I would like to help other teachers in their use of digital tools.	0	1	2	3	4	5	6	7
I have a very limited knowledge of digital tools.	0	1	2	3	4	5	6	7

I would like to know the effect of the reorganization of my teaching styles to accommodate digital tools on my professional status	0 1 2 3 4 5 6 7
I am concerned about conflict between my interests and my responsibilities.	0 1 2 3 4 5 6 7
I am concerned about revising my use of digital tools.	0 1 2 3 4 5 6 7
I would like to develop working relationships with both teachers in my school and teachers outside on the use of digital tools.	0 1 2 3 4 5 6 7
I am concerned about how digital tools affects students.	0 1 2 3 4 5 6 7
I am not concerned about digital tools at this time.	0 1 2 3 4 5 6 7
I would like to know who will make the decisions about the use of digital tools.	0 1 2 3 4 5 6 7
I would like to discuss the possibility of using digital tools	0 1 2 3 4 5 6 7
I would like to know what resources are available if we decide to adopt digital tools.	0 1 2 3 4 5 6 7
I am concerned about my inability to manage all that digital tools require.	0 1 2 3 4 5 6 7
I would like to know how my teaching or administration is supposed to change.	0 1 2 3 4 5 6 7
I would like to familiarize other co teachers with the progress of this new approach.	0 1 2 3 4 5 6 7
I am concerned about evaluating my impact on students.	0 1 2 3 4 5 6 7
I would like to revise the digital tools's approach.	0 1 2 3 4 5 6 7
I am preoccupied with things other than digital tools.	0 1 2 3 4 5 6 7
I would like to modify our use of digital tools based on the experiences of my students.	0 1 2 3 4 5 6 7
I spend little time thinking about digital tools.	0 1 2 3 4 5 6 7

I would like to excite my students about their part in this new approach.	0 1 2 3 4 5 6 7
I am concerned about time spent working with non educational problems related to the use of digital tools.	0 1 2 3 4 5 6 7
I would like to know what the use of digital tools would require in the immediate future.	0 1 2 3 4 5 6 7
I would like to coordinate my efforts with others to maximize the effects of digital tools in the classrooms.	0 1 2 3 4 5 6 7
I would like to have more information on time and energy commitments required by the use of digital tools	0 1 2 3 4 5 6 7
I would like to know what other colleagues are doing in this area.	0 1 2 3 4 5 6 7
Currently, other priorities prevent me from focusing my attention on the use of digital tools.	0 1 2 3 4 5 6 7
I would like to determine how to supplement, enhance, or replace digital tools	0 1 2 3 4 5 6 7
I would like to use feedback from students to modify the use of digital tools.	0 1 2 3 4 5 6 7
I would like to know how my role will change when I am using digital tools.	0 1 2 3 4 5 6 7
Coordination of tasks and pupils is taking too much of my time.	0 1 2 3 4 5 6 7
I would like to know how the use of digital tools is a better method than what we do now.	0 1 2 3 4 5 6 7

36. I am a Male Female
37. I have been teaching for <1 year 1-5years 6-10years 11-20years over 20years
38. I am ________ years old <20 years 20-30years 31-40years 41-50years over 50years
39. How good are you at using digital devices Very good good moderate poor No ability
40. My school is a Public School Private School
41. I teach Primary classes Secondary classes
42. My subject area is ________________ (e.g Mathematics)
